# Tris(dicyclo­hexyl­ammonium) hydrogen [1-hy­droxy-2-(1*H*-imidazol-1-yl)-1-phospho­natoethane]­phospho­nate ethanol monosolvate mono­hydrate

**DOI:** 10.1107/S1600536811042206

**Published:** 2011-10-22

**Authors:** Anindita Sarkar, Ignacy Cukrowski

**Affiliations:** a Department of Chemistry, University of Pretoria, Lynnwood Road, Pretoria 0002, South Africa

## Abstract

In the title compound, 3C_12_H_24_N^+^·C_5_H_7_N_2_O_7_P_2_^3−^·C_2_H_6_O·H_2_O, the zoledronic acid mol­ecule is singly protonated and stabilized by an intra­molecular O—H⋯O inter­action. The three-dimensional crystal structure is stabilized by inter­molecular O—H⋯O, O—H⋯N and N—H⋯O inter­actions. The ethanol solvent mol­ecule is disordered over two positions; the site-occupancy factor of the major component is 0.510 (4).

## Related literature

For the structure of zoledronic acid, see: Sanders *et al.* (2003 ▶); Ruscica *et al.* (2010 ▶).
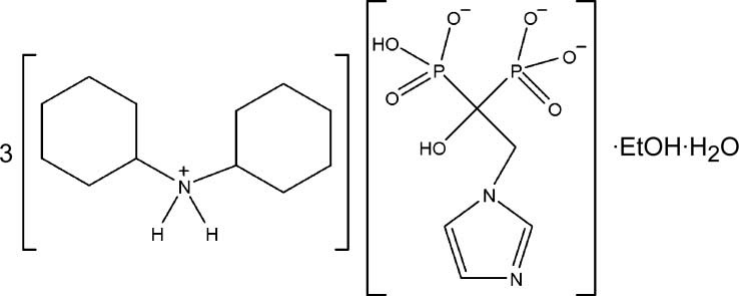


## Experimental

### 

#### Crystal data

3C_12_H_24_N^+^·C_5_H_7_N_2_O_7_P_2_^3−^·C_2_H_6_O·H_2_O
*M* *_r_* = 880.11
Triclinic, 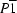

*a* = 14.2351 (2) Å
*b* = 14.3010 (3) Å
*c* = 15.3021 (3) Å
α = 64.626 (1)°
β = 79.725 (1)°
γ = 60.960 (1)°
*V* = 2459.04 (8) Å^3^
*Z* = 2
Mo *K*α radiation
μ = 0.14 mm^−1^
*T* = 173 K
0.42 × 0.32 × 0.29 mm

#### Data collection

Bruker APEXII CCD diffractometer
Absorption correction: multi-scan (*SADABS*; Bruker, 2001 ▶) *T*_min_ = 0.942, *T*_max_ = 0.960
24772 measured reflections
11852 independent reflections
9043 reflections with *I* > 2σ(*I*)
*R*_int_ = 0.035

#### Refinement

*R*[*F* ^2^ > 2σ(*F* ^2^)] = 0.039
*wR*(*F* ^2^) = 0.113
*S* = 1.06
11852 reflections
573 parameters
H atoms treated by a mixture of independent and constrained refinement
Δρ_max_ = 0.19 e Å^−3^
Δρ_min_ = −0.20 e Å^−3^

### 

Data collection: *APEX2* (Bruker, 2001 ▶); cell refinement: *SAINT* (Bruker, 2001 ▶); data reduction: *SAINT*; program(s) used to solve structure: *SHELXTL* (Sheldrick, 2008 ▶); program(s) used to refine structure: *SHELXL97* (Sheldrick, 2008 ▶); molecular graphics: *ORTEP-3 for Windows* (Farrugia, 1997 ▶) and *Mercury* (Bruno *et al.*, 2002 ▶); software used to prepare material for publication: *SHELXL97* and *PLATON* (Spek, 2009 ▶).

## Supplementary Material

Crystal structure: contains datablock(s) I, global. DOI: 10.1107/S1600536811042206/tk2800sup1.cif

Structure factors: contains datablock(s) I. DOI: 10.1107/S1600536811042206/tk2800Isup2.hkl

Additional supplementary materials:  crystallographic information; 3D view; checkCIF report

## Figures and Tables

**Table 1:** Hydrogen-bond geometry (Å, °)

*D*—H⋯*A*	*D*—H	H⋯*A*	*D*⋯*A*	*D*—H⋯*A*
O1*W*—H1*WA*⋯O7^i^	0.86 (2)	2.03 (2)	2.8725 (16)	168 (2)
O1*W*—H1*WB*⋯N4^ii^	0.86 (2)	2.16 (2)	2.9962 (18)	166 (2)
N3—H3*A*⋯O2^ii^	0.92	1.74	2.6291 (14)	163
N3—H3*B*⋯O3^iii^	0.92	1.84	2.7446 (14)	167
N1—H1*C*⋯O1^ii^	0.92	1.86	2.7543 (14)	163
N1—H1*D*⋯O7^ii^	0.92	1.88	2.7525 (15)	157
N2—H2*C*⋯O6^iii^	0.92	2.13	2.9580 (14)	150
N2—H2*C*⋯O5^iii^	0.92	2.39	3.1869 (15)	145
N2—H2*D*⋯O6^iv^	0.92	1.73	2.6522 (15)	177
O5—H5⋯O3	0.84	1.66	2.4871 (13)	167

Symmetry codes: (i) 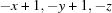; (ii) 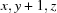; (iii) 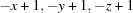; (iv) 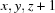.

**Fig. 1.**
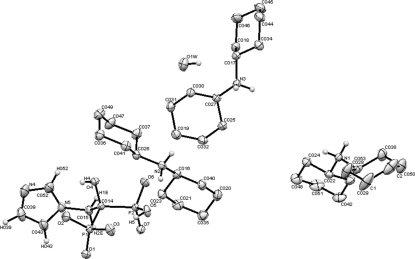
Molecular structure with atom labels and 50% displacement ellipsoids. The C—H atom of the cyclohexyl groups and the disordered ethanol molecule have been omitted for reasons of clarity.

**Fig. 2.**
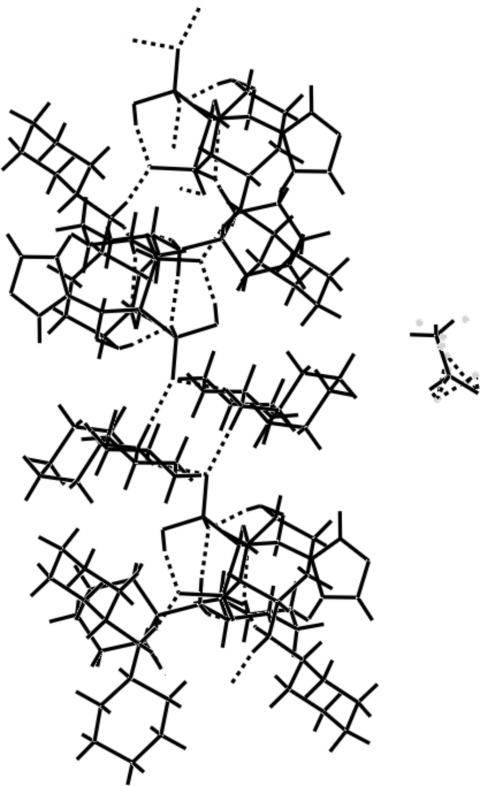
Hydrogen-bonded network of the molecule. Hydrogen bonds are shown as dashed lines.

## supplementary crystallographic information

### Comment

The asymmetric unit of the title compound contains three protonated dicyclohexylammine cations which act as counter ions to the negatively charged (-3) zoledronic acid (ZA) molecule, a water molecule and one distorted ethanol molecule. Each of the cyclohexyl rings adopt a typical chair conformation. The crystal packing is dominated by extensive hydrogen bonding which involves all O-atoms of the ZA anion, N—H···H atoms of the DCHA cations as well as O-atoms of the water molecule, Table 1 and Fig. 2. Each DCHA molecule has a different set of N—H hydrogen bonding interactions: (i) the N1—H atoms of one DCHA forms two H-bonds with O atoms of two phosphonate groups belonging to a single ZA molecule, (ii) the N2—H atoms form H-bonds with the same phosphonate group but of two different ZA molecules, and (iii) the N3—H atoms of third DCHA forms two H-bonds with O atoms of two different phosphonate groups belonging to different ZA molecule. It is likely that the disordered hydorxy group of the ethanol molecules forms O—H···O hydrogen bonds but, the O—H atom was not located in the study. One phosphonate group of zoledronic acid has an O atom that is protonated (O5) with the remaining five O atoms unprotonated. The P–O bond distances are comparable with the structure of zoledronic acid reported earlier (Ruscica *et al.*, 2010; Sanders *et al.*, 2003).

### Experimental

Dicyclohexylamine (0.26 ml, 2.484 *M*) was mixed with zoledronic acid (0.070 g, 0.26 mmol) in ethanol (1 ml) and water (0.5 ml). Colourless crystals were obtained after 15 days.

### Refinement

With the exception of water-H, the O-, N- and C-bound H atoms were positioned geometrically (O—H = 0.84 Å, N—H = 0.92 Å and C—H = 0.98-1.00 Å) and refined using a riding model with *U*_iso_(H) = 1.2–1.5*U*_eq_(O,N,C). The water-bound H atoms were refined with O—H = 0.84±0.02 Å and with no restraint on *U*_iso_(H). The solvent ethanol molecule was disordered over two positions. From anisotropic refinement, the major component had a site occupancy factor = 0.510 (4).

### Crystal data

**Table:**

3C_12_H_24_N^+^·C_5_H_7_N_2_O_7_P_2_^3^^−^·C_2_H_6_O·H_2_O	*Z* = 2
*M**_r_* = 880.11	*F*(000) = 962.0
Triclinic, *P*1	*D*_x_ = 1.187 Mg m^−^^3^
Hall symbol: -P 1	Mo *K*α radiation, λ = 0.71073 Å
*a* = 14.2351 (2) Å	Cell parameters from 9929 reflections
*b* = 14.3010 (3) Å	θ = 2.2–28.4°
*c* = 15.3021 (3) Å	µ = 0.14 mm^−^^1^
α = 64.626 (1)°	*T* = 173 K
β = 79.725 (1)°	Block, colourless
γ = 60.960 (1)°	0.42 × 0.32 × 0.29 mm
*V* = 2459.04 (8) Å^3^	

### Data collection

**Table:**

Bruker APEXII CCD diffractometer	11852 independent reflections
Radiation source: fine-focus sealed tube	9043 reflections with *I* > 2σ(*I*)
graphite	*R*_int_ = 0.035
φ and ω scans	θ_max_ = 28.0°, θ_min_ = 1.5°
Absorption correction: multi-scan (*SADABS*; Bruker, 2001)	*h* = −18→18
*T*_min_ = 0.942, *T*_max_ = 0.960	*k* = −18→16
24772 measured reflections	*l* = −20→11

### Refinement

**Table:**

Refinement on *F*^2^	Primary atom site location: structure-invariant direct methods
Least-squares matrix: full	Secondary atom site location: difference Fourier map
*R*[*F*^2^ > 2σ(*F*^2^)] = 0.039	Hydrogen site location: inferred from neighbouring sites
*wR*(*F*^2^) = 0.113	H atoms treated by a mixture of independent and constrained refinement
*S* = 1.06	*w* = 1/[σ^2^(*F*_o_^2^) + (0.064*P*)^2^] where *P* = (*F*_o_^2^ + 2*F*_c_^2^)/3
11852 reflections	(Δ/σ)_max_ < 0.001
573 parameters	Δρ_max_ = 0.19 e Å^−^^3^
0 restraints	Δρ_min_ = −0.20 e Å^−^^3^

### Special details

**Table:**

Geometry. All e.s.d.'s (except the e.s.d. in the dihedral angle between two l.s. planes) are estimated using the full covariance matrix. The cell e.s.d.'s are taken into account individually in the estimation of e.s.d.'s in distances, angles and torsion angles; correlations between e.s.d.'s in cell parameters are only used when they are defined by crystal symmetry. An approximate (isotropic) treatment of cell e.s.d.'s is used for estimating e.s.d.'s involving l.s. planes.
Refinement. Refinement of *F*^2^ against ALL reflections. The weighted *R*-factor *wR* and goodness of fit *S* are based on *F*^2^, conventional *R*-factors *R* are based on *F*, with *F* set to zero for negative *F*^2^. The threshold expression of *F*^2^ > σ(*F*^2^) is used only for calculating *R*-factors(gt) *etc*. and is not relevant to the choice of reflections for refinement. *R*-factors based on *F*^2^ are statistically about twice as large as those based on *F*, and *R*- factors based on ALL data will be even larger.

### Fractional atomic coordinates and isotropic or equivalent isotropic displacement parameters (Å^2^)

**Table:**

	*x*	*y*	*z*	*U*_iso_*/*U*_eq_	Occ. (<1)
C90A	0.8255 (8)	0.6106 (5)	0.3879 (5)	0.094 (3)	0.490 (4)
H90A	0.8721	0.5978	0.3336	0.112*	0.490 (4)
H90B	0.7513	0.6471	0.3623	0.112*	0.490 (4)
C91A	0.8440 (14)	0.4922 (11)	0.4541 (7)	0.078 (3)	0.490 (4)
H91A	0.9132	0.4354	0.4412	0.117*	0.490 (4)
H91B	0.7863	0.4787	0.4442	0.117*	0.490 (4)
H91C	0.8448	0.4834	0.5211	0.117*	0.490 (4)
O8A	0.8304 (2)	0.6892 (2)	0.39172 (18)	0.0507 (9)	0.490 (4)
C90B	0.7862 (5)	0.6122 (5)	0.4234 (4)	0.0614 (13)	0.510 (4)
H90C	0.7420	0.6005	0.4811	0.074*	0.510 (4)
H90D	0.8257	0.6472	0.4346	0.074*	0.510 (4)
C91B	0.8714 (16)	0.4931 (12)	0.4480 (11)	0.127 (6)	0.510 (4)
H91D	0.8398	0.4414	0.4596	0.191*	0.510 (4)
H91E	0.9112	0.4680	0.5066	0.191*	0.510 (4)
H91F	0.9205	0.4896	0.3944	0.191*	0.510 (4)
O8B	0.7226 (3)	0.6925 (3)	0.3688 (4)	0.129 (2)	0.510 (4)
C2	1.00873 (15)	1.0093 (2)	0.36588 (14)	0.0587 (6)	
H2A	0.9368	1.0724	0.3680	0.070*	
H2B	1.0608	1.0093	0.4010	0.070*	
C1	1.00647 (16)	0.8941 (2)	0.41524 (13)	0.0650 (7)	
H1A	0.9835	0.8826	0.4825	0.078*	
H1B	1.0798	0.8305	0.4179	0.078*	
H1WA	0.2343 (16)	0.8889 (18)	0.0453 (16)	0.062 (6)*	
H1WB	0.2395 (18)	0.951 (2)	0.0943 (16)	0.070 (7)*	
P1	0.60569 (3)	0.02501 (3)	0.29745 (2)	0.01979 (8)	
P2	0.64304 (3)	0.21333 (3)	0.11618 (2)	0.01986 (8)	
O1	0.69397 (7)	−0.08702 (8)	0.29121 (6)	0.0253 (2)	
O7	0.74110 (7)	0.13417 (8)	0.08033 (7)	0.0259 (2)	
O4	0.45160 (7)	0.22357 (8)	0.17711 (7)	0.0264 (2)	
H4	0.4229	0.1934	0.2265	0.040*	
N3	0.35697 (8)	0.98062 (9)	0.46030 (8)	0.0212 (2)	
H3A	0.4138	0.9935	0.4292	0.025*	
H3B	0.3639	0.9648	0.5244	0.025*	
N1	0.88010 (8)	0.91338 (10)	0.20255 (8)	0.0229 (2)	
H1C	0.8260	0.9054	0.2432	0.028*	
H1D	0.8495	0.9845	0.1514	0.028*	
O6	0.57731 (8)	0.33379 (8)	0.04307 (7)	0.0296 (2)	
N2	0.45671 (9)	0.49208 (9)	0.88322 (8)	0.0228 (2)	
H2C	0.4311	0.5639	0.8850	0.027*	
H2D	0.4967	0.4372	0.9396	0.027*	
O2	0.50436 (8)	0.02028 (9)	0.34226 (7)	0.0335 (2)	
N5	0.48470 (9)	0.03465 (10)	0.12324 (8)	0.0258 (2)	
O5	0.67158 (8)	0.23054 (9)	0.20118 (7)	0.0294 (2)	
H5	0.6656	0.1816	0.2545	0.044*	
O3	0.64782 (9)	0.07412 (9)	0.34427 (6)	0.0309 (2)	
C014	0.55908 (10)	0.13700 (10)	0.17132 (9)	0.0185 (2)	
C015	0.55684 (11)	0.08745 (12)	0.10003 (9)	0.0237 (3)	
H1E	0.5358	0.1513	0.0348	0.028*	
H2E	0.6307	0.0280	0.0969	0.028*	
C016	0.53088 (11)	0.48714 (12)	0.80079 (9)	0.0240 (3)	
H016	0.4902	0.5518	0.7395	0.029*	
C017	0.25503 (10)	1.09136 (11)	0.41869 (10)	0.0242 (3)	
H017	0.1940	1.0812	0.4582	0.029*	
C018	0.23269 (12)	1.11974 (12)	0.31394 (10)	0.0299 (3)	
H01A	0.2212	1.0578	0.3117	0.036*	
H01B	0.2958	1.1218	0.2752	0.036*	
C019	0.41039 (12)	0.63145 (12)	0.53018 (11)	0.0306 (3)	
H01C	0.4167	0.5670	0.5166	0.037*	
H01D	0.4252	0.6016	0.6004	0.037*	
C020	0.70241 (13)	0.49679 (14)	0.73781 (11)	0.0349 (3)	
H02A	0.6668	0.5606	0.6753	0.042*	
H02B	0.7622	0.5059	0.7516	0.042*	
C021	0.65676 (12)	0.36045 (14)	0.71381 (11)	0.0341 (3)	
H02C	0.6874	0.2818	0.7136	0.041*	
H02D	0.6211	0.4184	0.6497	0.041*	
C022	0.92751 (10)	0.81937 (11)	0.16445 (10)	0.0243 (3)	
H022	0.9696	0.7431	0.2184	0.029*	
C023	0.57366 (12)	0.37133 (12)	0.79166 (11)	0.0292 (3)	
H02E	0.6068	0.3065	0.8545	0.035*	
H02F	0.5132	0.3651	0.7753	0.035*	
C024	0.83706 (11)	0.81334 (12)	0.13088 (10)	0.0282 (3)	
H02G	0.7929	0.8891	0.0789	0.034*	
H02H	0.7901	0.7977	0.1855	0.034*	
C025	0.48111 (11)	0.77654 (12)	0.48583 (11)	0.0296 (3)	
H02I	0.4955	0.7557	0.5544	0.035*	
H02J	0.5335	0.8026	0.4459	0.035*	
C026	0.36177 (11)	0.47296 (12)	0.88350 (9)	0.0239 (3)	
H026	0.3893	0.3904	0.8921	0.029*	
C027	0.36713 (10)	0.87424 (11)	0.45431 (9)	0.0223 (3)	
H027	0.3545	0.8930	0.3852	0.027*	
C028	0.95888 (11)	0.91368 (12)	0.25662 (10)	0.0265 (3)	
H028	1.0319	0.8502	0.2548	0.032*	
C029	1.00309 (11)	0.84036 (13)	0.08306 (11)	0.0309 (3)	
H02K	1.0624	0.8417	0.1071	0.037*	
H02L	0.9635	0.9168	0.0297	0.037*	
C030	0.28512 (11)	0.83668 (12)	0.51468 (10)	0.0273 (3)	
H03A	0.2117	0.9013	0.4931	0.033*	
H03B	0.2962	0.8176	0.5835	0.033*	
C031	0.29686 (12)	0.73023 (13)	0.50407 (11)	0.0295 (3)	
H03C	0.2461	0.7036	0.5467	0.035*	
H03D	0.2781	0.7522	0.4364	0.035*	
C032	0.49365 (12)	0.67013 (12)	0.47371 (11)	0.0330 (3)	
H03E	0.4854	0.6896	0.4042	0.040*	
H03F	0.5667	0.6053	0.4968	0.040*	
N4	0.34581 (12)	0.01110 (13)	0.10898 (11)	0.0448 (3)	
C034	0.26366 (12)	1.18894 (12)	0.42759 (10)	0.0292 (3)	
H03G	0.3275	1.1957	0.3935	0.035*	
H03H	0.2732	1.1700	0.4967	0.035*	
C035	0.74712 (12)	0.37986 (14)	0.73014 (12)	0.0357 (3)	
H03I	0.7965	0.3778	0.6757	0.043*	
H03J	0.7887	0.3164	0.7904	0.043*	
C036	0.19760 (12)	0.47029 (13)	0.97509 (11)	0.0320 (3)	
H03K	0.2218	0.3877	0.9883	0.038*	
H03L	0.1528	0.4873	1.0290	0.038*	
C037	0.29459 (12)	0.55261 (14)	0.78926 (10)	0.0307 (3)	
H03M	0.3391	0.5371	0.7348	0.037*	
H03N	0.2697	0.6350	0.7773	0.037*	
C038	0.96294 (14)	1.02890 (15)	0.20716 (12)	0.0385 (4)	
H03O	0.8900	1.0935	0.2035	0.046*	
H03P	0.9871	1.0398	0.1403	0.046*	
C039	0.41881 (15)	−0.08851 (15)	0.17662 (12)	0.0415 (4)	
H039	0.4105	−0.1570	0.2122	0.050*	
C040	0.62157 (12)	0.50537 (13)	0.81803 (10)	0.0287 (3)	
H04A	0.6585	0.4457	0.8813	0.034*	
H04B	0.5915	0.5830	0.8200	0.034*	
C041	0.29558 (11)	0.48930 (13)	0.97021 (10)	0.0280 (3)	
H04C	0.2715	0.5691	0.9653	0.034*	
H04D	0.3407	0.4333	1.0303	0.034*	
C042	1.04932 (13)	0.74347 (15)	0.04539 (12)	0.0410 (4)	
H04E	1.0959	0.7594	−0.0094	0.049*	
H04F	1.0940	0.6680	0.0973	0.049*	
C043	0.50512 (14)	−0.07631 (13)	0.18620 (12)	0.0376 (4)	
H043	0.5668	−0.1331	0.2280	0.045*	
C044	0.16307 (13)	1.30485 (13)	0.38436 (12)	0.0381 (4)	
H04G	0.1002	1.3002	0.4221	0.046*	
H04H	0.1717	1.3674	0.3886	0.046*	
C045	0.14310 (15)	1.33456 (13)	0.27923 (13)	0.0427 (4)	
H04I	0.2032	1.3457	0.2403	0.051*	
H04J	0.0761	1.4084	0.2536	0.051*	
C046	0.13349 (14)	1.23724 (13)	0.27022 (13)	0.0422 (4)	
H04K	0.1243	1.2563	0.2010	0.051*	
H04L	0.0689	1.2318	0.3037	0.051*	
C047	0.19748 (12)	0.53181 (15)	0.79479 (11)	0.0363 (3)	
H04M	0.1526	0.5867	0.7344	0.044*	
H04N	0.2227	0.4515	0.8004	0.044*	
C048	0.88304 (14)	0.71705 (14)	0.09334 (12)	0.0394 (4)	
H04O	0.8236	0.7162	0.0691	0.047*	
H04P	0.9218	0.6406	0.1470	0.047*	
C049	0.13026 (12)	0.54850 (15)	0.88050 (11)	0.0364 (3)	
H04Q	0.0706	0.5302	0.8841	0.044*	
H04R	0.0986	0.6310	0.8714	0.044*	
C050	1.03987 (15)	1.03247 (18)	0.26256 (13)	0.0484 (4)	
H05A	1.1142	0.9731	0.2604	0.058*	
H05B	1.0381	1.1100	0.2313	0.058*	
C051	0.95965 (14)	0.73577 (16)	0.01224 (13)	0.0458 (4)	
H05C	0.9912	0.6704	−0.0089	0.055*	
H05D	0.9193	0.8086	−0.0438	0.055*	
C052	0.38893 (13)	0.08289 (14)	0.07855 (12)	0.0388 (4)	
H052	0.3560	0.1600	0.0305	0.047*	
C053	0.92903 (14)	0.88867 (19)	0.36123 (11)	0.0493 (5)	
H05E	0.9326	0.8103	0.3925	0.059*	
H05F	0.8544	0.9466	0.3645	0.059*	
O1W	0.21111 (9)	0.91039 (11)	0.09257 (9)	0.0379 (3)	

### Atomic displacement parameters (Å^2^)

**Table:**

	*U*^11^	*U*^22^	*U*^33^	*U*^12^	*U*^13^	*U*^23^
C90A	0.148 (7)	0.037 (3)	0.060 (4)	−0.032 (4)	0.042 (4)	−0.016 (3)
C91A	0.100 (7)	0.041 (4)	0.051 (4)	−0.019 (4)	0.002 (3)	0.002 (3)
O8A	0.0571 (18)	0.0344 (15)	0.0500 (16)	−0.0172 (13)	−0.0102 (13)	−0.0076 (11)
C90B	0.064 (3)	0.048 (3)	0.053 (3)	−0.023 (3)	0.001 (2)	−0.006 (2)
C91B	0.135 (12)	0.064 (6)	0.171 (11)	−0.030 (6)	0.020 (7)	−0.061 (7)
O8B	0.060 (2)	0.043 (2)	0.222 (5)	−0.0197 (18)	0.049 (3)	−0.021 (3)
C2	0.0382 (10)	0.1057 (18)	0.0635 (12)	−0.0349 (11)	0.0051 (9)	−0.0593 (13)
C1	0.0457 (11)	0.131 (2)	0.0322 (9)	−0.0528 (13)	0.0015 (8)	−0.0297 (11)
P1	0.02154 (17)	0.01921 (16)	0.01897 (16)	−0.01042 (13)	0.00183 (13)	−0.00707 (13)
P2	0.02148 (17)	0.01830 (16)	0.02077 (16)	−0.01085 (13)	0.00050 (13)	−0.00648 (13)
O1	0.0240 (5)	0.0208 (5)	0.0269 (5)	−0.0079 (4)	−0.0009 (4)	−0.0079 (4)
O7	0.0235 (5)	0.0263 (5)	0.0304 (5)	−0.0133 (4)	0.0042 (4)	−0.0125 (4)
O4	0.0176 (4)	0.0212 (5)	0.0343 (5)	−0.0062 (4)	0.0019 (4)	−0.0093 (4)
N3	0.0202 (5)	0.0229 (5)	0.0206 (5)	−0.0111 (5)	0.0020 (4)	−0.0080 (4)
N1	0.0181 (5)	0.0244 (6)	0.0285 (6)	−0.0093 (5)	−0.0002 (4)	−0.0124 (5)
O6	0.0303 (5)	0.0220 (5)	0.0299 (5)	−0.0127 (4)	−0.0020 (4)	−0.0027 (4)
N2	0.0257 (6)	0.0218 (5)	0.0234 (5)	−0.0106 (5)	−0.0033 (4)	−0.0099 (4)
O2	0.0274 (5)	0.0326 (6)	0.0329 (5)	−0.0158 (5)	0.0100 (4)	−0.0078 (4)
N5	0.0284 (6)	0.0266 (6)	0.0279 (6)	−0.0162 (5)	−0.0036 (5)	−0.0096 (5)
O5	0.0419 (6)	0.0334 (5)	0.0261 (5)	−0.0270 (5)	0.0023 (4)	−0.0119 (4)
O3	0.0488 (6)	0.0313 (5)	0.0208 (5)	−0.0243 (5)	−0.0025 (4)	−0.0089 (4)
C014	0.0164 (6)	0.0170 (6)	0.0220 (6)	−0.0073 (5)	−0.0004 (5)	−0.0075 (5)
C015	0.0272 (7)	0.0249 (7)	0.0236 (6)	−0.0156 (6)	−0.0005 (5)	−0.0089 (5)
C016	0.0272 (7)	0.0243 (6)	0.0223 (6)	−0.0121 (6)	−0.0018 (5)	−0.0093 (5)
C017	0.0196 (6)	0.0221 (6)	0.0284 (7)	−0.0092 (5)	0.0022 (5)	−0.0088 (5)
C018	0.0292 (7)	0.0278 (7)	0.0344 (8)	−0.0134 (6)	−0.0065 (6)	−0.0105 (6)
C019	0.0353 (8)	0.0259 (7)	0.0322 (7)	−0.0156 (6)	−0.0010 (6)	−0.0101 (6)
C020	0.0340 (8)	0.0396 (9)	0.0364 (8)	−0.0235 (7)	0.0033 (7)	−0.0125 (7)
C021	0.0361 (8)	0.0365 (8)	0.0338 (8)	−0.0147 (7)	0.0042 (6)	−0.0209 (7)
C022	0.0222 (7)	0.0215 (6)	0.0278 (7)	−0.0073 (5)	0.0003 (5)	−0.0114 (5)
C023	0.0308 (7)	0.0297 (7)	0.0350 (8)	−0.0151 (6)	0.0025 (6)	−0.0186 (6)
C024	0.0280 (7)	0.0301 (7)	0.0316 (7)	−0.0138 (6)	0.0029 (6)	−0.0166 (6)
C025	0.0228 (7)	0.0252 (7)	0.0371 (8)	−0.0105 (6)	0.0010 (6)	−0.0099 (6)
C026	0.0259 (7)	0.0245 (7)	0.0258 (7)	−0.0123 (6)	−0.0010 (5)	−0.0121 (5)
C027	0.0241 (7)	0.0214 (6)	0.0212 (6)	−0.0102 (5)	0.0013 (5)	−0.0088 (5)
C028	0.0175 (6)	0.0335 (7)	0.0319 (7)	−0.0103 (6)	−0.0022 (5)	−0.0164 (6)
C029	0.0235 (7)	0.0315 (8)	0.0330 (8)	−0.0099 (6)	0.0041 (6)	−0.0132 (6)
C030	0.0246 (7)	0.0285 (7)	0.0337 (7)	−0.0145 (6)	0.0043 (6)	−0.0152 (6)
C031	0.0307 (8)	0.0316 (7)	0.0333 (7)	−0.0178 (6)	0.0001 (6)	−0.0144 (6)
C032	0.0285 (8)	0.0236 (7)	0.0407 (8)	−0.0085 (6)	0.0038 (6)	−0.0125 (6)
N4	0.0416 (8)	0.0516 (9)	0.0569 (9)	−0.0303 (7)	−0.0054 (7)	−0.0218 (7)
C034	0.0346 (8)	0.0271 (7)	0.0283 (7)	−0.0150 (6)	0.0006 (6)	−0.0120 (6)
C035	0.0308 (8)	0.0393 (8)	0.0348 (8)	−0.0156 (7)	0.0054 (6)	−0.0151 (7)
C036	0.0313 (8)	0.0366 (8)	0.0325 (8)	−0.0173 (7)	0.0054 (6)	−0.0170 (7)
C037	0.0309 (8)	0.0398 (8)	0.0245 (7)	−0.0183 (7)	−0.0019 (6)	−0.0117 (6)
C038	0.0429 (9)	0.0447 (9)	0.0413 (9)	−0.0278 (8)	−0.0055 (7)	−0.0168 (7)
C039	0.0564 (11)	0.0434 (9)	0.0430 (9)	−0.0363 (9)	0.0009 (8)	−0.0168 (8)
C040	0.0328 (8)	0.0293 (7)	0.0305 (7)	−0.0181 (6)	−0.0014 (6)	−0.0119 (6)
C041	0.0311 (7)	0.0308 (7)	0.0254 (7)	−0.0144 (6)	0.0011 (6)	−0.0139 (6)
C042	0.0322 (8)	0.0414 (9)	0.0411 (9)	−0.0071 (7)	0.0084 (7)	−0.0237 (8)
C043	0.0444 (9)	0.0291 (8)	0.0422 (9)	−0.0216 (7)	−0.0108 (7)	−0.0064 (7)
C044	0.0403 (9)	0.0245 (7)	0.0458 (9)	−0.0130 (7)	0.0030 (7)	−0.0137 (7)
C045	0.0462 (10)	0.0240 (8)	0.0494 (10)	−0.0114 (7)	−0.0155 (8)	−0.0062 (7)
C046	0.0394 (9)	0.0294 (8)	0.0519 (10)	−0.0111 (7)	−0.0200 (8)	−0.0085 (7)
C047	0.0301 (8)	0.0511 (10)	0.0347 (8)	−0.0198 (7)	−0.0030 (6)	−0.0201 (7)
C048	0.0424 (9)	0.0352 (8)	0.0492 (10)	−0.0151 (7)	−0.0011 (8)	−0.0262 (8)
C049	0.0270 (8)	0.0453 (9)	0.0402 (9)	−0.0156 (7)	0.0020 (7)	−0.0213 (7)
C050	0.0486 (10)	0.0653 (12)	0.0567 (11)	−0.0368 (10)	−0.0028 (9)	−0.0316 (10)
C051	0.0453 (10)	0.0484 (10)	0.0449 (10)	−0.0099 (8)	0.0037 (8)	−0.0341 (8)
C052	0.0360 (9)	0.0363 (9)	0.0474 (9)	−0.0186 (7)	−0.0142 (7)	−0.0110 (7)
C053	0.0409 (10)	0.0849 (14)	0.0293 (8)	−0.0406 (10)	−0.0014 (7)	−0.0141 (9)
O1W	0.0382 (6)	0.0469 (7)	0.0386 (6)	−0.0257 (6)	0.0036 (5)	−0.0190 (6)

### Geometric parameters (Å, °)

**Table:**

C90A—O8A	1.185 (7)	C024—H02G	0.9900
C90A—C91A	1.468 (13)	C024—H02H	0.9900
C90A—H90A	0.9900	C025—C027	1.5225 (18)
C90A—H90B	0.9900	C025—C032	1.5323 (19)
C91A—H91A	0.9800	C025—H02I	0.9900
C91A—H91B	0.9800	C025—H02J	0.9900
C91A—H91C	0.9800	C026—C041	1.5194 (18)
C90B—O8B	1.129 (6)	C026—C037	1.5226 (19)
C90B—C91B	1.457 (16)	C026—H026	1.0000
C90B—H90C	0.9900	C027—C030	1.5178 (17)
C90B—H90D	0.9900	C027—H027	1.0000
C91B—H91D	0.9800	C028—C053	1.512 (2)
C91B—H91E	0.9800	C028—C038	1.516 (2)
C91B—H91F	0.9800	C028—H028	1.0000
C2—C050	1.500 (3)	C029—C042	1.533 (2)
C2—C1	1.504 (3)	C029—H02K	0.9900
C2—H2A	0.9900	C029—H02L	0.9900
C2—H2B	0.9900	C030—C031	1.5255 (18)
C1—C053	1.539 (2)	C030—H03A	0.9900
C1—H1A	0.9900	C030—H03B	0.9900
C1—H1B	0.9900	C031—H03C	0.9900
P1—O2	1.5025 (10)	C031—H03D	0.9900
P1—O1	1.5128 (9)	C032—H03E	0.9900
P1—O3	1.5361 (10)	C032—H03F	0.9900
P1—C014	1.8726 (13)	N4—C052	1.322 (2)
P2—O6	1.5006 (10)	N4—C039	1.365 (2)
P2—O7	1.5020 (9)	C034—C044	1.526 (2)
P2—O5	1.5740 (9)	C034—H03G	0.9900
P2—C014	1.8572 (12)	C034—H03H	0.9900
O4—C014	1.4451 (14)	C035—H03I	0.9900
O4—H4	0.8400	C035—H03J	0.9900
N3—C027	1.4983 (16)	C036—C049	1.527 (2)
N3—C017	1.5011 (16)	C036—C041	1.529 (2)
N3—H3A	0.9200	C036—H03K	0.9900
N3—H3B	0.9200	C036—H03L	0.9900
N1—C022	1.5012 (16)	C037—C047	1.531 (2)
N1—C028	1.5103 (16)	C037—H03M	0.9900
N1—H1C	0.9200	C037—H03N	0.9900
N1—H1D	0.9200	C038—C050	1.531 (2)
N2—C016	1.4964 (16)	C038—H03O	0.9900
N2—C026	1.5023 (16)	C038—H03P	0.9900
N2—H2C	0.9200	C039—C043	1.362 (2)
N2—H2D	0.9200	C039—H039	0.9500
N5—C052	1.3481 (19)	C040—H04A	0.9900
N5—C043	1.3684 (18)	C040—H04B	0.9900
N5—C015	1.4641 (16)	C041—H04C	0.9900
O5—H5	0.8400	C041—H04D	0.9900
C014—C015	1.5459 (17)	C042—C051	1.521 (2)
C015—H1E	0.9900	C042—H04E	0.9900
C015—H2E	0.9900	C042—H04F	0.9900
C016—C040	1.5203 (19)	C043—H043	0.9500
C016—C023	1.5239 (18)	C044—C045	1.520 (2)
C016—H016	1.0000	C044—H04G	0.9900
C017—C034	1.5219 (18)	C044—H04H	0.9900
C017—C018	1.5278 (19)	C045—C046	1.526 (2)
C017—H017	1.0000	C045—H04I	0.9900
C018—C046	1.528 (2)	C045—H04J	0.9900
C018—H01A	0.9900	C046—H04K	0.9900
C018—H01B	0.9900	C046—H04L	0.9900
C019—C031	1.516 (2)	C047—C049	1.514 (2)
C019—C032	1.523 (2)	C047—H04M	0.9900
C019—H01C	0.9900	C047—H04N	0.9900
C019—H01D	0.9900	C048—C051	1.522 (2)
C020—C035	1.524 (2)	C048—H04O	0.9900
C020—C040	1.528 (2)	C048—H04P	0.9900
C020—H02A	0.9900	C049—H04Q	0.9900
C020—H02B	0.9900	C049—H04R	0.9900
C021—C035	1.521 (2)	C050—H05A	0.9900
C021—C023	1.5232 (19)	C050—H05B	0.9900
C021—H02C	0.9900	C051—H05C	0.9900
C021—H02D	0.9900	C051—H05D	0.9900
C022—C029	1.5212 (18)	C052—H052	0.9500
C022—C024	1.5201 (19)	C053—H05E	0.9900
C022—H022	1.0000	C053—H05F	0.9900
C023—H02E	0.9900	O1W—H1WA	0.86 (2)
C023—H02F	0.9900	O1W—H1WB	0.86 (2)
C024—C048	1.5242 (19)		
			
O8A—C90A—C91A	137.3 (9)	N3—C027—H027	108.2
O8A—C90A—H90A	102.8	C030—C027—H027	108.2
C91A—C90A—H90A	102.8	C025—C027—H027	108.2
O8A—C90A—H90B	102.8	N1—C028—C053	110.09 (11)
C91A—C90A—H90B	102.8	N1—C028—C038	109.98 (11)
H90A—C90A—H90B	105.0	C053—C028—C038	111.91 (13)
O8B—C90B—C91B	147.7 (10)	N1—C028—H028	108.3
O8B—C90B—H90C	99.8	C053—C028—H028	108.3
C91B—C90B—H90C	99.8	C038—C028—H028	108.3
O8B—C90B—H90D	99.8	C022—C029—C042	109.83 (12)
C91B—C90B—H90D	99.8	C022—C029—H02K	109.7
H90C—C90B—H90D	104.1	C042—C029—H02K	109.7
C90B—C91B—H91D	109.5	C022—C029—H02L	109.7
C90B—C91B—H91E	109.4	C042—C029—H02L	109.7
H91D—C91B—H91E	109.5	H02K—C029—H02L	108.2
C90B—C91B—H91F	109.5	C027—C030—C031	109.89 (11)
H91D—C91B—H91F	109.5	C027—C030—H03A	109.7
H91E—C91B—H91F	109.5	C031—C030—H03A	109.7
C050—C2—C1	111.36 (15)	C027—C030—H03B	109.7
C050—C2—H2A	109.4	C031—C030—H03B	109.7
C1—C2—H2A	109.4	H03A—C030—H03B	108.2
C050—C2—H2B	109.4	C019—C031—C030	111.56 (12)
C1—C2—H2B	109.4	C019—C031—H03C	109.3
H2A—C2—H2B	108.0	C030—C031—H03C	109.3
C2—C1—C053	111.20 (17)	C019—C031—H03D	109.3
C2—C1—H1A	109.4	C030—C031—H03D	109.3
C053—C1—H1A	109.4	H03C—C031—H03D	108.0
C2—C1—H1B	109.4	C019—C032—C025	111.22 (12)
C053—C1—H1B	109.4	C019—C032—H03E	109.4
H1A—C1—H1B	108.0	C025—C032—H03E	109.4
O2—P1—O1	115.51 (6)	C019—C032—H03F	109.4
O2—P1—O3	111.83 (6)	C025—C032—H03F	109.4
O1—P1—O3	111.41 (6)	H03E—C032—H03F	108.0
O2—P1—C014	102.98 (6)	C052—N4—C039	104.33 (13)
O1—P1—C014	108.19 (5)	C017—C034—C044	110.88 (12)
O3—P1—C014	106.07 (5)	C017—C034—H03G	109.5
O6—P2—O7	116.63 (6)	C044—C034—H03G	109.5
O6—P2—O5	105.70 (6)	C017—C034—H03H	109.5
O7—P2—O5	112.01 (6)	C044—C034—H03H	109.5
O6—P2—C014	110.28 (6)	H03G—C034—H03H	108.1
O7—P2—C014	106.76 (5)	C021—C035—C020	110.83 (12)
O5—P2—C014	104.88 (5)	C021—C035—H03I	109.5
C014—O4—H4	109.5	C020—C035—H03I	109.5
C027—N3—C017	116.91 (10)	C021—C035—H03J	109.5
C027—N3—H3A	108.1	C020—C035—H03J	109.5
C017—N3—H3A	108.1	H03I—C035—H03J	108.1
C027—N3—H3B	108.1	C049—C036—C041	111.24 (12)
C017—N3—H3B	108.1	C049—C036—H03K	109.4
H3A—N3—H3B	107.3	C041—C036—H03K	109.4
C022—N1—C028	114.95 (10)	C049—C036—H03L	109.4
C022—N1—H1C	108.5	C041—C036—H03L	109.4
C028—N1—H1C	108.5	H03K—C036—H03L	108.0
C022—N1—H1D	108.5	C026—C037—C047	110.10 (12)
C028—N1—H1D	108.5	C026—C037—H03M	109.6
H1C—N1—H1D	107.5	C047—C037—H03M	109.6
C016—N2—C026	117.97 (10)	C026—C037—H03N	109.6
C016—N2—H2C	107.8	C047—C037—H03N	109.6
C026—N2—H2C	107.8	H03M—C037—H03N	108.2
C016—N2—H2D	107.8	C028—C038—C050	110.84 (14)
C026—N2—H2D	107.8	C028—C038—H03O	109.5
H2C—N2—H2D	107.2	C050—C038—H03O	109.5
C052—N5—C043	106.44 (13)	C028—C038—H03P	109.5
C052—N5—C015	125.90 (12)	C050—C038—H03P	109.5
C043—N5—C015	127.33 (12)	H03O—C038—H03P	108.1
P2—O5—H5	109.5	C043—C039—N4	110.79 (14)
O4—C014—C015	108.99 (10)	C043—C039—H039	124.6
O4—C014—P2	107.37 (8)	N4—C039—H039	124.6
C015—C014—P2	106.20 (8)	C016—C040—C020	110.64 (11)
O4—C014—P1	107.01 (8)	C016—C040—H04A	109.5
C015—C014—P1	113.89 (8)	C020—C040—H04A	109.5
P2—C014—P1	113.16 (6)	C016—C040—H04B	109.5
N5—C015—C014	115.88 (10)	C020—C040—H04B	109.5
N5—C015—H1E	108.3	H04A—C040—H04B	108.1
C014—C015—H1E	108.3	C026—C041—C036	110.83 (11)
N5—C015—H2E	108.3	C026—C041—H04C	109.5
C014—C015—H2E	108.3	C036—C041—H04C	109.5
H1E—C015—H2E	107.4	C026—C041—H04D	109.5
N2—C016—C040	108.23 (10)	C036—C041—H04D	109.5
N2—C016—C023	110.60 (10)	H04C—C041—H04D	108.1
C040—C016—C023	111.51 (11)	C051—C042—C029	110.81 (13)
N2—C016—H016	108.8	C051—C042—H04E	109.5
C040—C016—H016	108.8	C029—C042—H04E	109.5
C023—C016—H016	108.8	C051—C042—H04F	109.5
N3—C017—C034	108.35 (11)	C029—C042—H04F	109.5
N3—C017—C018	111.67 (10)	H04E—C042—H04F	108.1
C034—C017—C018	111.70 (11)	C039—C043—N5	105.86 (14)
N3—C017—H017	108.3	C039—C043—H043	127.1
C034—C017—H017	108.3	N5—C043—H043	127.1
C018—C017—H017	108.3	C045—C044—C034	111.04 (12)
C017—C018—C046	111.01 (12)	C045—C044—H04G	109.4
C017—C018—H01A	109.4	C034—C044—H04G	109.4
C046—C018—H01A	109.4	C045—C044—H04H	109.4
C017—C018—H01B	109.4	C034—C044—H04H	109.4
C046—C018—H01B	109.4	H04G—C044—H04H	108.0
H01A—C018—H01B	108.0	C044—C045—C046	110.53 (13)
C031—C019—C032	111.96 (12)	C044—C045—H04I	109.5
C031—C019—H01C	109.2	C046—C045—H04I	109.5
C032—C019—H01C	109.2	C044—C045—H04J	109.5
C031—C019—H01D	109.2	C046—C045—H04J	109.5
C032—C019—H01D	109.2	H04I—C045—H04J	108.1
H01C—C019—H01D	107.9	C045—C046—C018	111.64 (13)
C035—C020—C040	110.94 (12)	C045—C046—H04K	109.3
C035—C020—H02A	109.5	C018—C046—H04K	109.3
C040—C020—H02A	109.5	C045—C046—H04L	109.3
C035—C020—H02B	109.5	C018—C046—H04L	109.3
C040—C020—H02B	109.5	H04K—C046—H04L	108.0
H02A—C020—H02B	108.0	C049—C047—C037	111.37 (12)
C035—C021—C023	111.89 (12)	C049—C047—H04M	109.4
C035—C021—H02C	109.2	C037—C047—H04M	109.4
C023—C021—H02C	109.2	C049—C047—H04N	109.4
C035—C021—H02D	109.2	C037—C047—H04N	109.4
C023—C021—H02D	109.2	H04M—C047—H04N	108.0
H02C—C021—H02D	107.9	C024—C048—C051	110.80 (13)
N1—C022—C029	111.20 (11)	C024—C048—H04O	109.5
N1—C022—C024	109.14 (10)	C051—C048—H04O	109.5
C029—C022—C024	111.21 (11)	C024—C048—H04P	109.5
N1—C022—H022	108.4	C051—C048—H04P	109.5
C029—C022—H022	108.4	H04O—C048—H04P	108.1
C024—C022—H022	108.4	C047—C049—C036	111.34 (13)
C021—C023—C016	111.31 (12)	C047—C049—H04Q	109.4
C021—C023—H02E	109.4	C036—C049—H04Q	109.4
C016—C023—H02E	109.4	C047—C049—H04R	109.4
C021—C023—H02F	109.4	C036—C049—H04R	109.4
C016—C023—H02F	109.4	H04Q—C049—H04R	108.0
H02E—C023—H02F	108.0	C2—C050—C038	110.59 (14)
C022—C024—C048	110.21 (12)	C2—C050—H05A	109.5
C022—C024—H02G	109.6	C038—C050—H05A	109.5
C048—C024—H02G	109.6	C2—C050—H05B	109.5
C022—C024—H02H	109.6	C038—C050—H05B	109.5
C048—C024—H02H	109.6	H05A—C050—H05B	108.1
H02G—C024—H02H	108.1	C042—C051—C048	111.05 (13)
C027—C025—C032	109.15 (12)	C042—C051—H05C	109.4
C027—C025—H02I	109.9	C048—C051—H05C	109.4
C032—C025—H02I	109.9	C042—C051—H05D	109.4
C027—C025—H02J	109.9	C048—C051—H05D	109.4
C032—C025—H02J	109.9	H05C—C051—H05D	108.0
H02I—C025—H02J	108.3	N4—C052—N5	112.58 (15)
N2—C026—C041	108.16 (10)	N4—C052—H052	123.7
N2—C026—C037	112.42 (11)	N5—C052—H052	123.7
C041—C026—C037	111.72 (12)	C028—C053—C1	110.06 (13)
N2—C026—H026	108.1	C028—C053—H05E	109.6
C041—C026—H026	108.1	C1—C053—H05E	109.6
C037—C026—H026	108.1	C028—C053—H05F	109.6
N3—C027—C030	111.94 (10)	C1—C053—H05F	109.6
N3—C027—C025	109.10 (11)	H05E—C053—H05F	108.2
C030—C027—C025	110.96 (11)	H1WA—O1W—H1WB	110.9 (19)
			
C050—C2—C1—C053	−57.3 (2)	N1—C022—C029—C042	−179.58 (12)
O6—P2—C014—O4	−33.95 (10)	C024—C022—C029—C042	−57.72 (15)
O7—P2—C014—O4	−161.57 (8)	N3—C027—C030—C031	178.53 (11)
O5—P2—C014—O4	79.42 (9)	C025—C027—C030—C031	−59.34 (15)
O6—P2—C014—C015	82.53 (9)	C032—C019—C031—C030	−53.61 (16)
O7—P2—C014—C015	−45.08 (10)	C027—C030—C031—C019	55.65 (15)
O5—P2—C014—C015	−164.10 (8)	C031—C019—C032—C025	54.24 (17)
O6—P2—C014—P1	−151.81 (6)	C027—C025—C032—C019	−56.60 (16)
O7—P2—C014—P1	80.58 (7)	N3—C017—C034—C044	178.75 (11)
O5—P2—C014—P1	−38.44 (8)	C018—C017—C034—C044	55.33 (15)
O2—P1—C014—O4	34.80 (9)	C023—C021—C035—C020	−55.03 (17)
O1—P1—C014—O4	157.54 (8)	C040—C020—C035—C021	56.37 (17)
O3—P1—C014—O4	−82.82 (9)	N2—C026—C037—C047	−178.37 (11)
O2—P1—C014—C015	−85.71 (10)	C041—C026—C037—C047	−56.55 (15)
O1—P1—C014—C015	37.03 (10)	N1—C028—C038—C050	178.30 (12)
O3—P1—C014—C015	156.67 (9)	C053—C028—C038—C050	55.60 (18)
O2—P1—C014—P2	152.86 (7)	C052—N4—C039—C043	−0.22 (19)
O1—P1—C014—P2	−84.39 (7)	N2—C016—C040—C020	177.93 (11)
O3—P1—C014—P2	35.25 (8)	C023—C016—C040—C020	56.06 (15)
C052—N5—C015—C014	104.47 (16)	C035—C020—C040—C016	−56.98 (16)
C043—N5—C015—C014	−83.02 (17)	N2—C026—C041—C036	−179.68 (11)
O4—C014—C015—N5	−57.82 (14)	C037—C026—C041—C036	56.07 (15)
P2—C014—C015—N5	−173.21 (9)	C049—C036—C041—C026	−54.71 (16)
P1—C014—C015—N5	61.58 (13)	C022—C029—C042—C051	56.69 (17)
C026—N2—C016—C040	−178.36 (11)	N4—C039—C043—N5	0.56 (19)
C026—N2—C016—C023	−55.93 (15)	C052—N5—C043—C039	−0.67 (17)
C027—N3—C017—C034	−177.16 (10)	C015—N5—C043—C039	−174.35 (13)
C027—N3—C017—C018	−53.73 (14)	C017—C034—C044—C045	−56.82 (17)
N3—C017—C018—C046	−175.66 (11)	C034—C044—C045—C046	57.03 (18)
C034—C017—C018—C046	−54.15 (16)	C044—C045—C046—C018	−56.10 (19)
C028—N1—C022—C029	−68.64 (14)	C017—C018—C046—C045	54.53 (18)
C028—N1—C022—C024	168.31 (11)	C026—C037—C047—C049	56.32 (17)
C035—C021—C023—C016	54.06 (17)	C022—C024—C048—C051	−56.70 (17)
N2—C016—C023—C021	−175.05 (11)	C037—C047—C049—C036	−55.94 (18)
C040—C016—C023—C021	−54.56 (15)	C041—C036—C049—C047	54.97 (17)
N1—C022—C024—C048	−179.06 (12)	C1—C2—C050—C038	57.1 (2)
C029—C022—C024—C048	57.90 (15)	C028—C038—C050—C2	−55.9 (2)
C016—N2—C026—C041	−176.64 (11)	C029—C042—C051—C048	−56.45 (18)
C016—N2—C026—C037	−52.81 (15)	C024—C048—C051—C042	56.40 (18)
C017—N3—C027—C030	−65.27 (14)	C039—N4—C052—N5	−0.23 (19)
C017—N3—C027—C025	171.53 (10)	C043—N5—C052—N4	0.58 (19)
C032—C025—C027—N3	−176.50 (10)	C015—N5—C052—N4	174.37 (13)
C032—C025—C027—C030	59.72 (14)	N1—C028—C053—C1	−177.60 (16)
C022—N1—C028—C053	−115.98 (14)	C038—C028—C053—C1	−55.0 (2)
C022—N1—C028—C038	120.26 (13)	C2—C1—C053—C028	55.5 (2)

### Hydrogen-bond geometry (Å, °)

**Table:**

*D*—H···*A*	*D*—H	H···*A*	*D*···*A*	*D*—H···*A*
O1W—H1WA···O7^i^	0.86 (2)	2.03 (2)	2.8725 (16)	168 (2)
O1W—H1WB···N4^ii^	0.86 (2)	2.16 (2)	2.9962 (18)	166 (2)
N3—H3A···O2^ii^	0.92	1.74	2.6291 (14)	163
N3—H3B···O3^iii^	0.92	1.84	2.7446 (14)	167
N1—H1C···O1^ii^	0.92	1.86	2.7543 (14)	163
N1—H1D···O7^ii^	0.92	1.88	2.7525 (15)	157
N2—H2C···O6^iii^	0.92	2.13	2.9580 (14)	150
N2—H2C···O5^iii^	0.92	2.39	3.1869 (15)	145
N2—H2D···O6^iv^	0.92	1.73	2.6522 (15)	177
O5—H5···O3	0.84	1.66	2.4871 (13)	167

Symmetry codes: (i) −*x*+1, −*y*+1, −*z*; (ii) *x*, *y*+1, *z*; (iii) −*x*+1, −*y*+1, −*z*+1; (iv) *x*, *y*, *z*+1.
